# Supplementary material for: Integrating ensemble systems biology feature selection and bimodal deep neural network for breast cancer prognosis prediction
Source: Sci Rep. 2021 Jul 21;11:14914. doi: 10.1038/s41598-021-92864-y (PMC8295302; doi:10.1038/s41598-021-92864-y)
Supplement: Supplementary file 1 — Supplementary Information. [file 41598_2021_92864_MOESM1_ESM.pdf]

Supplementary Information

Integrating ensemble systems biology feature selection and bimodal deep neural network for breast cancer prognosis prediction

Li-Hsin Cheng, Te-Cheng Hsu, Che Lin

A. Data preprocessing and data distribution

There are 24,338 available gene features and 10 relevant clinical features in the METABRIC dataset.

The gene features are preprocessed microarray gene expression values. Although gene expression measured through the RNA-Seq technique is the major trend nowadays, which contains lower background noise, METABRIC is the largest available open-access breast cancer cohort containing gene expression, clinical information, and long-term survival data that allows relevant data analysis. We did not use a combined dataset by merging multiple small cohorts, since different platforms, sample curation workflows, and experimental procedures can all lead to different measurement outputs. Even if multiple cohorts are combined after careful preprocessing and standardization, it is still difficult to obtain robust feature selection and prognosis prediction results using such a combined dataset with heterogeneous composition.

The clinical features were derived from the raw clinical information in the dataset, which include age, tumor size, neoplasm histologic grade, cellularity, menopausal state, radio therapy, chemotherapy, hormone therapy, breast conserving surgery, and breast mastectomy surgery. Since most features (the latter six) are binary, we normalized the other features (the first four) according to the training data, ensuring that the scale of clinical features did not differ significantly from each other.

After excluding samples with missing values, the dataset was divided into an unlabeled, training, and hold-out testing sets. Table S1 provides a summary of the three subsets. Furthermore, we summarized the clinical feature distributions for each class (poor/good prognosis) in both the training and testing sets, summarized in Table S2.

	Unlabeled	Training	Testing
**Samples**	1282	465	117
**Class**			
Good prognosis	-	221 (48%)	55 (47%)
Poor prognosis	-	244 (52%)	62 (53%)
**Median DSS time (months)**	-	56.33	56.27
**Median age (years)**	61.99	61.55	59.82
**Median tumor size (mm)**	21	26	25
**ER status**			
Positive	1023 (80%)	326 (70%)	81 (69%)
Negative	259 (20%)	139 (30%)	36 (31%)
**PR status**			
Positive	729 (57%)	207 (45%)	54 (46%)
Negative	553 (43%)	258 (55%)	63 (54%)
**HER2 status**			
Positive	120 (9%)	86 (18%)	23 (20%)
Negative	1162 (91%)	379 (82%)	94 (80%)
**Menopausal state**			
Pre	260 (20%)	105 (23%)	32 (27%)
Post	1022 (80%)	360 (77%)	85 (73%)
**Neoplasm Histologic Grade**			
1	140 (11%)	16 (3%)	7 (6%)
2	530 (43%)	164 (35%)	38 (32%)
3	556 (45%)	285 (61%)	72 (62%)
**Cellularity**			
Low	142 (11%)	40 (9%)	15 (13%)
Moderate	474 (38%)	173 (37%)	50 (43%)
High	626 (50%)	252 (54%)	52 (44%)

Table S1. Data distribution overview

	Training	Training	Testing	Testing
**Prognosis class**	Poor	Good	Poor	Good
**Samples**	244	221	62	55
**Median DSS time (months)**	35.22	108.07	32.33	110.83
**Median age (years)**	59.88	62.00	57.84	60.98
**Median tumor size (mm)**	30.00	25.00	28.50	20.00
**ER status**				
Positive	131 (54%)	195 (88%)	33 (53%)	48 (87%)
Negative	113 (46%)	26 (12%)	29 (47%)	7 (13%)
**PR status**				
Positive	74 (30%)	133 (60%)	20 (32%)	34 (62%)
Negative	170 (70%)	88 (40%)	42 (68%)	21 (38%)
**HER2 status**				
Positive	60 (25%)	26 (12%)	12 (19%)	11 (20%)
Negative	184 (75%)	195 (88%)	50 (81%)	44 (80%)
**Menopausal state**				
Pre	63 (26%)	42 (19%)	21 (34%)	11 (20%)
Post	181 (74%)	179 (81%)	41 (66%)	44 (80%)
**Neoplasm Histologic Grade**				
1	10 (4%)	6 (3%)	1 (2%)	6 (11%)
2	61 (25%)	103 (47%)	13 (21%)	25 (45%)
3	173 (71%)	112 (50%)	48 (77%)	24 (44%)
**Cellularity**				
Low	22 (9%)	18 (8%)	7 (11%)	8 (15%)
Moderate	82 (34%)	91 (41%)	25 (40%)	25 (45%)
High	140 (57%)	112 (51%)	30 (49%)	22 (40%)

Table S2. Clinical feature distribution overview

B. Systems biology feature selector

The inputted samples were divided into two groups according to the binary split criterion that was assigned, for example, ER+ samples and ER- samples. Genes without significant differential expression between the two groups were excluded by ANOVA. Next, we constructed interaction networks for each group based on the interaction information documented in the BioGRID database. We used gene expression data to estimate the interaction ability between genes and excluded false positive links. The constructed interaction networks would therefore be disease-specific and tailored for the inputted group of samples.

The main assumption of the network construction method is that the expression level of a gene is affected by other genes, which can thus be represented by the linear combination of the expression level of its interaction partners:

$$x_i[n] = \sum_{j \in \mathrm{G}_i} a_{ij} x_j[n] + \varepsilon_i[n] \tag{1}$$

where $x_i[n]$ is the expression level of gene i for patient n; a_{ij} is the interaction ability between genes i and j; G_i is the set of genes that are related to gene i according to BioGRID; and $\varepsilon_i[n]$ is stochastic noise. Equation (1) can be rewritten into the matrix form:

$$\boldsymbol{X} = \boldsymbol{A}\boldsymbol{X} + \boldsymbol{E} \tag{2}$$

where

$$\boldsymbol{X} = \begin{bmatrix} x_1[1] & \cdots & x_1[N] \\ \vdots & \ddots & \vdots \\ x_M[1] & \cdots & x_M[N] \end{bmatrix},\ \boldsymbol{A} = \begin{bmatrix} a_{11} & \cdots & a_{1M} \\ \vdots & a_{ij} & \vdots \\ a_{M1} & \cdots & a_{MM} \end{bmatrix},\ \boldsymbol{E} = \begin{bmatrix} \varepsilon_1[1] & \cdots & \varepsilon_1[N] \\ \vdots & \ddots & \vdots \\ \varepsilon_M[1] & \cdots & \varepsilon_M[N] \end{bmatrix}$$

where M is the number of genes left after excluding those without differential expression by ANOVA, and N is the sample size. The interaction abilities were estimated by Linear Minimum Mean Square Error (LMMSE). Afterwards, we performed model selection and excluded false positive interactions through Akaike information criterion (AIC) and *t*-test. If a_{ij} is not equal to a_{ji}, we took the one with the larger absolute value as the final interaction ability between genes i and j. After calculating all the interaction abilities, we then obtain the final interaction ability matrix A. By constructing interaction networks for two sample groups (e.g., ER+ samples and ER- samples), we would get A^+ and A^- for each group. We define the difference matrix D to be:

$$\boldsymbol{D} = \boldsymbol{A}^{+} - \boldsymbol{A}^{-} = \begin{bmatrix} d_{11} & \cdots & d_{1M} \\ \vdots & \ddots & \vdots \\ d_{M1} & \cdots & d_{MM} \end{bmatrix} = \begin{bmatrix} a_{11}^{+} - a_{11}^{-} & \cdots & a_{1M}^{+} - a_{1M}^{-} \\ \vdots & \ddots & \vdots \\ a_{M1}^{+} - a_{M1}^{-} & \cdots & a_{MM}^{+} - a_{MM}^{-} \end{bmatrix} \quad (3)$$

where d_{ij} is the difference in interaction ability between genes i and j. The prognosis relevance value (PRV) is then defined as:

$$PRV_i = \sum_{j=1}^{M} |d_{ij}| \quad (4)$$

which is the summarized interaction ability difference between gene i and its interaction partners. For each gene, a higher PRV implies greater difference in its interaction abilities between two networks. Since the two networks represent different prognosis statuses, genes with high PRVs can be selected as potential prognosis biomarkers, which serve as an extension to the original inputted prognosis-relevant split criterion.

C. Bimodal DNN

Figure S1 illustrates the structure of bimodal DNN. The bimodal DNN processes gene expression input and clinical input with two separated subnetworks. The output of two subnetworks were then merged together, processed by successive hidden layers, and then turned into a final prediction output. The weights of the subnetworks were pre-trained. During the first phase of training, we froze the weights of subnetworks and trained only the weights between the merged layer and final output. During the second phase of training, all weights were unfrozen to allow fine-tuning of the whole network.

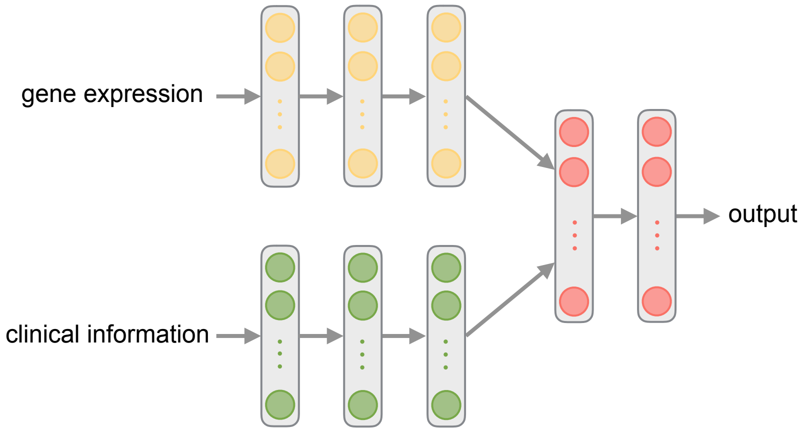

Fig. S1. Bimodal DNN model structure

D. Data perturbation ensemble approach

For the data perturbation random sampling setting, we tried subsampling 90%, 80%, and 70% of the whole data each time. In addition, we tried different number of subsamples: repeating 5, 10, 20, or 30 times. There were therefore 12 possible combinations of subsampling rates and number of subsamples. We evaluated the 12 settings through random validation by adding the areas of seven feature selectors to be the final summarized area.

From the comparison (Fig. S2), we found that all of the data perturbation results outperformed those of the original feature selection for all settings. Among all, subsampling 70% and repeating 5 times achieved the highest performance, which became the final data perturbation setting we used.

Ensemble learning approaches require a diverse set of base learners to cooperate such that the final prediction is strong and robust. Even with the subsampling rate set to 70%, candidate gene features frequently appear in most subsamples have a high chance of being selected as the final gene features. They may have significant biological meanings since they frequently achieved high PRV scores in such a set of high variety data subsamples. Moreover, given the marginal differences under 70% subsampling rate, a small ensemble is preferred due to its low computational complexity. As a result, we chose the 70%x5 setting that achieves both the best performance and the lowest complexity.

After determining the final data perturbation setting, we then compared the seven original feature selectors with their data-perturbation versions. From pairwise comparisons for each feature selector (Fig. S3), we found that data perturbation improves the robustness in most of the cases except for PR-selector. The improvement was verified through the one-tailed paired t-test, which implied that the "summarized area" distribution of the data-perturbation results for ER, HER2, TN, HP, MKI67, and PLAU-selectors were all significantly higher than their

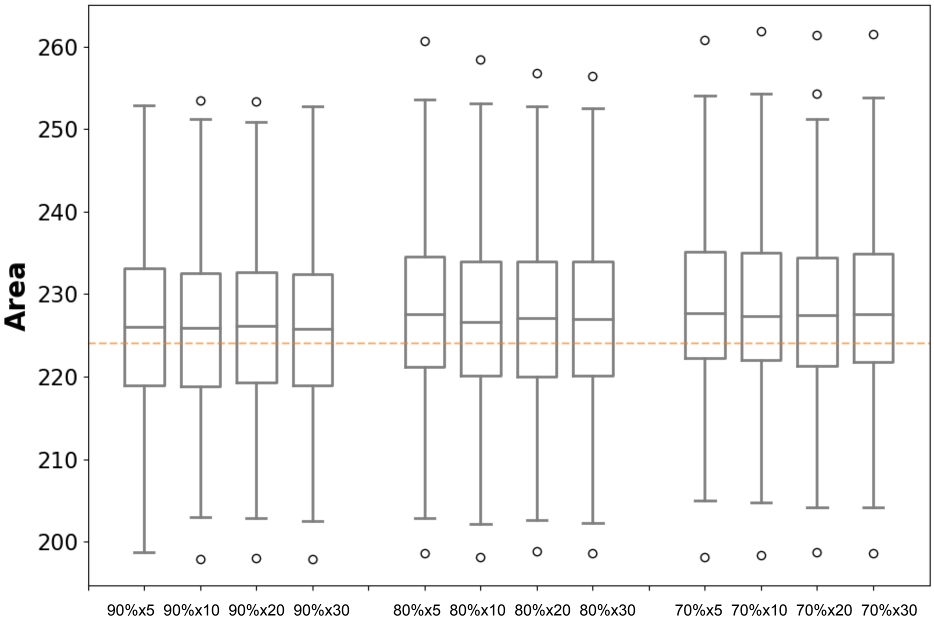

corresponding original feature selection results.

Fig. S2. Comparison of different data perturbation subsampling settings. The orange line represents the median of the original feature selection result.

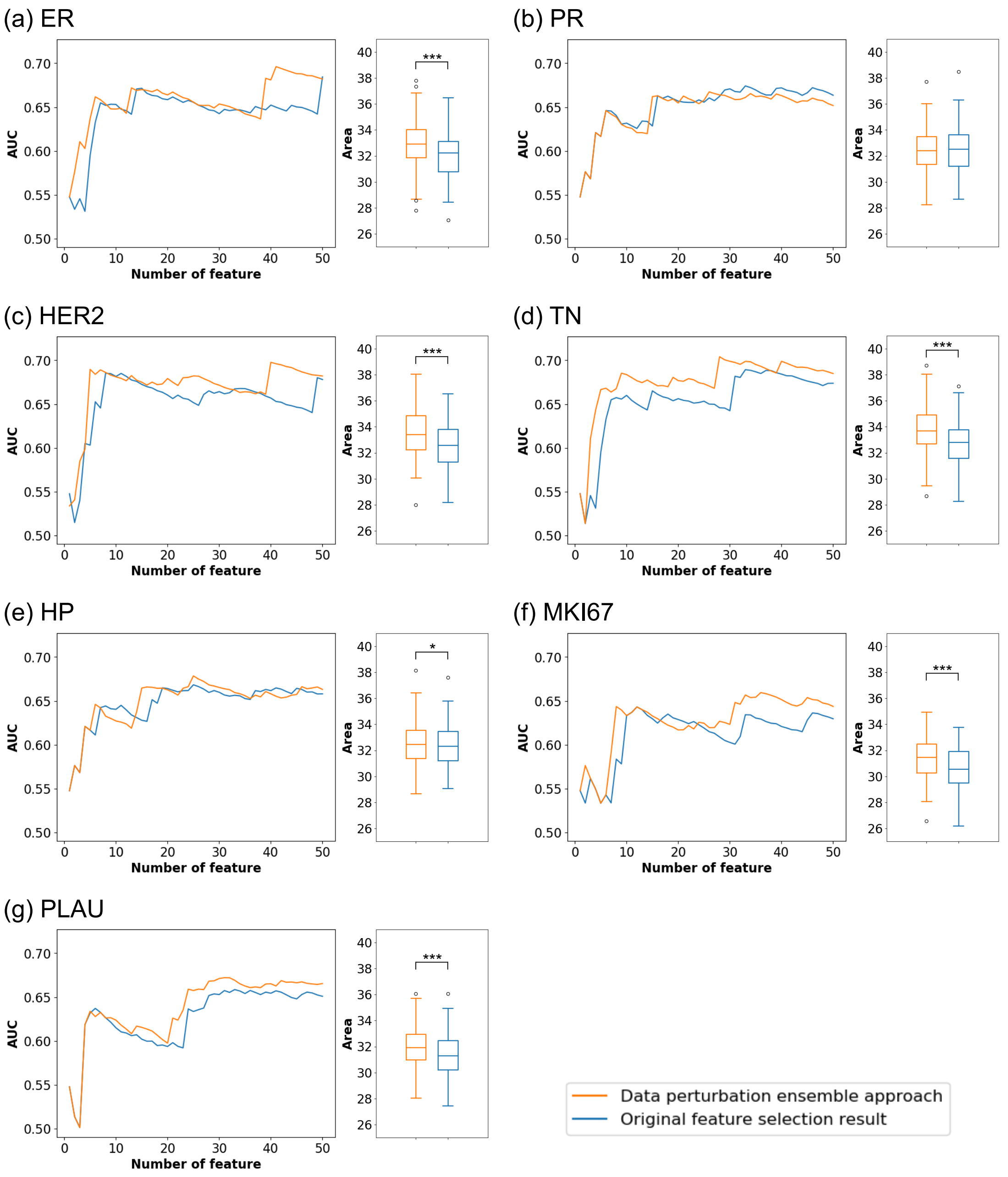

Fig. S3. Pairwise comparison of data perturbation and original feature selection results

E. Function perturbation ensemble approach

Since the output scale of different systems biology feature selection functions are different, we carefully evaluated the aggregation strategy when combining multiple PRV lists in function perturbation. The following are the aggregation strategies we tested:

(1) **PRV-median**: For each gene, take the median in all PRV lists as its final feature ranking score.
(2) **PRV-norm-median**: Normalize each component PRV list to 0–1. For each gene, take the median of all normalized PRV lists as its final feature ranking score.
(3) **PRV-mean**: For each gene, take the mean in all PRV lists as its final feature ranking score.
(4) **PRV-norm-mean**: Normalize each component PRV list to 0–1. For each gene, take the mean in all normalized PRV lists as its final feature ranking score.
(5) **Rank-median**: Transform each component PRV list into ranking list. For each gene, take the median ranking in all lists as its final feature ranking score.
(6) **Rank-mean**: Transform each component PRV list into ranking list. For each gene, take the mean ranking of all lists as its final feature ranking score.
(7) **Top N overlap**: Take the interaction of the top N genes from each list as the final selected gene. This aggregation strategy cannot output a final feature ranking score but just final selected genes.

Through random validation (Fig. S4) we found that rank-mean and top N overlap produce the best performance. However, since the top N overlap strategy cannot output a feature ranking score and the number of final selected genes cannot be determined by the user, there will be more limitation when using the top N overlap strategy in real applications. Therefore, we adopted rank-mean as our final aggregation strategy for function perturbation.

Having determined the aggregation strategy, we compared the original results of the seven feature selectors. Through Fig. S5, we found that function perturbation brought significant improvement to the original feature selection results, statistically verified by the one-tailed paired t-test.

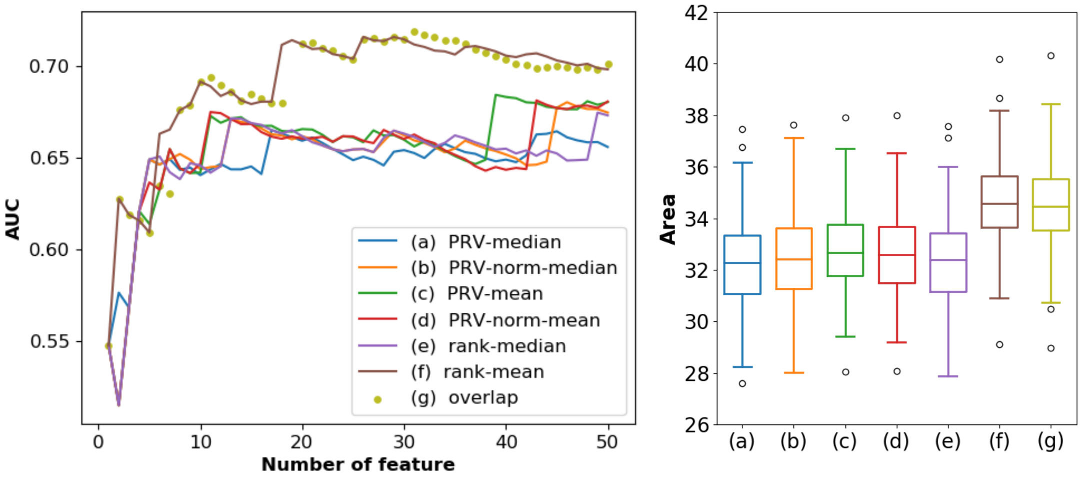

Fig. S4. Comparison of different function perturbation aggregation strategies

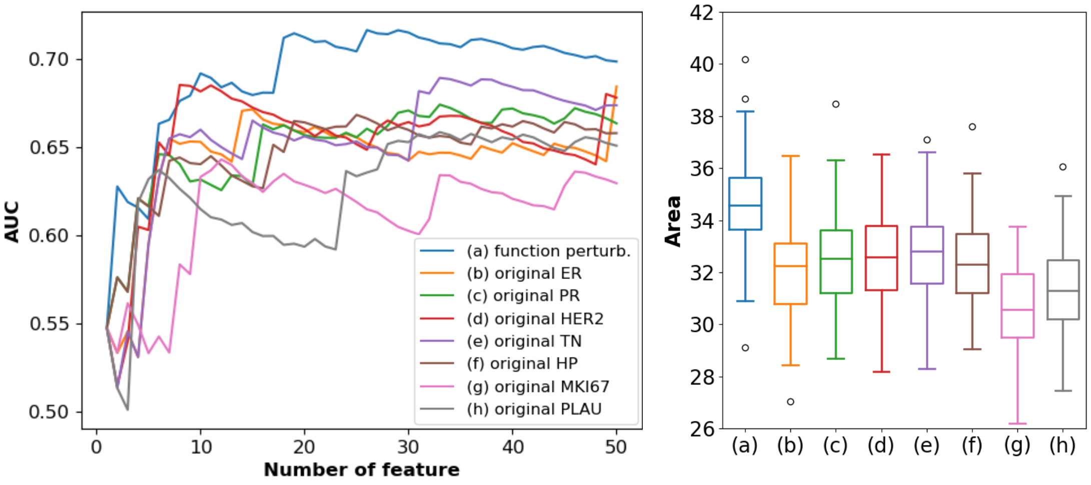

Fig. S5. Comparison of function perturbation and original feature selection results

F. The final selected gene set

The top 50 selected genes via the hybrid ensemble approach are listed below. The final selected genes are the first 16 genes that produced the peak performance.

1	ELAVL1	11	ZDHHC17	21	AURKA	31	CDK4	41	EIF4E2
2	EGFR	12	ENO1	22	ARRB1	32	SLC15A1	42	IGBP1
3	BTRC	13	DBN1	23	FLNA	33	MCM5	43	CDK1
4	FBXO6	14	PLK1	24	CREBBP	34	PPP2CA	44	CHEK1
5	SHMT2	15	ESR1	25	PCM1	35	TCTN1	45	CTDSPL
6	KRAS	16	GSK3B	26	ANLN	36	TENC1	46	ECT2
7	SRPK2	17	HIST1H3A	27	RPS14	37	KCTD3	47	AHSA1
8	YWHAQ	18	FBXW7	28	TRIM23	38	CENPJ	48	ACTR2
9	PDHA1	19	UCHL5	29	RPL6	39	HDAC11	49	POC5
10	EWSR1	20	SYNCRIP	30	TUBA1C	40	FUS	50	YBX1

Table S3. Top 50 selected genes via hybrid ensemble approach

We compared the performance with 100 random validation summarized areas among three gene sets, including the final feature selection result (16 genes), the top 50 selected genes in Table S3 (50 genes, including the final 16 genes), and all genes before feature selection (24,338 genes). A detailed box-plot illustrates the above results is available in Fig. S6. As illustrated in Fig. S6, we observed that the final 16 genes significantly outperform all genes, verified by the one-tailed paired *t*-test. We also observed that the top 50 selected genes performed inferior to the final selected genes with less median of summarized areas but still significantly outperformed all genes. As the gene set with the top 50 genes contains the final 16 genes, we do not expect them to perform much worse. Overall, we observed that the medians of the summarized areas among these three gene sets increased when fewer gene features were included. The corresponding distribution also became more concentrated. As fewer features reduce cost in clinical application and prevent model overfitting, we chose the final 16-gene set, which was then utilized for training our bimodal neural network classifier.

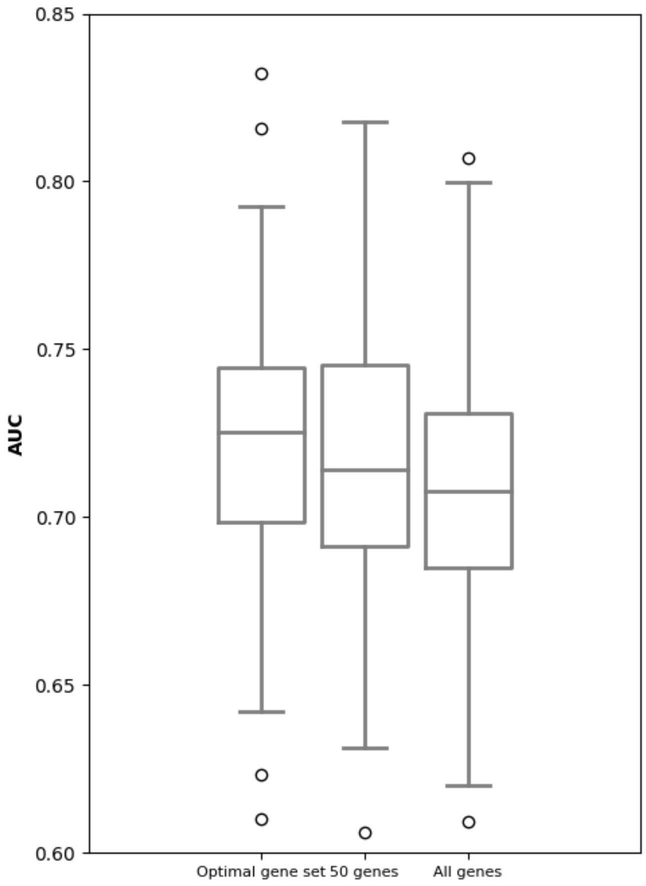

Fig. S6. Comparison of final genes (optimal gene set, 16 genes), top 50 genes, and all genes. Between the final 16 genes and the top 50 genes, the p-value of a one-tailed paired t-test is 0.094. Between the final 16 genes and all genes, the p-value of the one-tailed paired t-test is 0.001. Between the top 50 genes and all genes, the p-value of the one-tailed paired t-test is 0.007.

G. Hyperparameter grid search result

Below lists the hyperparameter grid search range for SVM, RF, and DNN. We searched through all possible combinations of the listed values. The final determined hyperparameter values are the combinations that achieved the best 4-fold cross validation performance, which are highlighted in bold.

	Gene feature SVM	Clinical feature SVM	Combined feature SVM
**C**	0.1, **1**, 10, 100	0.1, 1, 10, **100**	0.1, 1, **10**, 100
**gamma**	0.00001,0.00005,0.0001,<br>0.0005,0.001,0.005,0.01,**0.05**	0.00001,0.00005,0.0001,<br>0.0005,0.001,0.005,**0.01**,0.05	0.00001,0.00005,0.0001,<br>0.0005,**0.001**,0.005,0.01,0.05

Table S4. SVM hyperparameter grid search

	Gene feature RF	Clinical feature RF	Combined feature RF
**max_features**	4,**5**,6,7,8,9,10,11,12	4,5,6,**7**,8,9,10	4,5,6,7,8,**9**,10,11,12
**max_depth**	**4**,5,6,7,8,9,10,11,12	4,5,6,**7**,8,9,10	**4**,5,6,7,8,9,10,11,12
**min_samples_leaf**	**10**	**10**	**10**
**n_estimators**	**1000**	**1000**	**1000**

Table S5. RF hyperparameter grid search

	Gene feature DNN	Clinical feature DNN	Combining part of bimodal DNN
**number of hidden layer**	2, **3**	1, **2**	**1**
**number of neuron**	10, **20**	**5**, 10	5, **10**
**activation function**	**relu**, tanh	relu, **tanh**	relu, **tanh**
**learning rate**	0.002, **0.0002**	**0.002**, 0.0002, 0.00002	**0.00002**
**batch size**	**10**	**10**	**10**
**Number of epoch**	**200**, 400, 600, 800	200, 400, 600, **800**	epoch_1*: 50, **100**<br>epoch_2**: 0, 50, **100**
**optimizer**	**Nadam**	**Nadam**	**Nadam**
**l2-regularization term**	**0. 0001**	**0.0001**	**0.0001**
**dropout rate**	**0.05**	**0.05**	**0.05**
**max_norm constraint**	**1**	**1**	**1**

Table S6. DNN hyperparameter grid search

H. Test performance of selected genes as single biomarkers

Through test performance evaluation, we found that models with gene feature alone (Table 1a) can achieve an AUC between 0.7443 and 0.7672. This performance achieved by a multi-gene approach is higher than the AUC of any component gene as a single biomarker (Table S6). This indicates that a multi-gene approach can indeed model the complex molecular process of breast cancer more comprehensively through joint evaluation of multiple genes.

	**Positive / negative correlation with poor prognosis**	**AUC**	**CI**
**ELAVL1**	-	0.5471	0.5254
**EGFR**	+	0.6739	0.6170
**BTRC**	-	0.7258	0.6228
**FBXO6**	+	0.5169	0.5195
**SHMT2**	+	0.6957	0.6331
**KRAS**	+	0.6581	0.5894
**SRPK2**	-	0.7029	0.6078
**YWHAQ**	+	0.6575	0.6113
**PDHA1**	+	0.7032	0.6095
**EWSR1**	-	0.5386	0.5083
**ZDHHC17**	-	0.6507	0.5960
**ENO1**	+	0.6516	0.6036
**DBN1**	+	0.5809	0.5566
**PLK1**	+	0.7125	**0.6406**
**ESR1**	-	**0.7349**	0.6321
**GSK3B**	+	0.5836	0.5504
**PGR**	-	0.7062	0.6235
**ERBB2**	-	0.505	0.5035
**MKI67**	+	0.6982	0.6166
**PLAU**	+	0.6132	0.5520

Table S7. Test performance of selected genes as single biomarkers

I. Gene feature selection frequency curves

According to [1], we plotted the gene feature selection frequency curves for all function perturbation and the hybrid ensemble approaches, as illustrated in Fig. S7. The horizontal axis is the number of genes in the gene feature set. We have 5 subsample lists, each with 70% subsamples from data perturbation. The vertical axis is the maximum number of subsample lists that share the identical gene feature set. These curves examine the repeatability/robustness of our function perturbation and hybrid approaches by counting the frequency of the number of subsample lists that share the identical gene set with a certain number of genes. Each function perturbation approach can be considered as a gene feature selector with a well-known gene used as its split criterion. Hence, we have 7 function perturbation curves, one hybrid ensemble curve, and the ideal curve. The feature selector is considered robust by achieving high subsample counts since it produces similar gene feature sets for different subsample lists, which can be observed with larger areas under the curves.

Please note that for the hybrid ensemble approach in Fig. S7, the gene feature set was aggregated from the 7 function perturbation gene sets. In Fig. S7, the ideal case was plotted with the black dashed line, which indicates that one would obtain the exact same 50 genes in each subsample for a specific function perturbation approach. As illustrated in Fig. S7, the subsample lists of each function perturbation and the hybrid ensemble approaches have approximately 20 matched gene features. In particular, the hybrid ensemble approach achieved the most robust performance by selecting a maximum of 36 gene features matched in all 5 subsample lists as marked in red. The final 16 genes for building our bimodal breast cancer prognosis prediction model were further selected from the aggregated hybrid ensemble list (Fig. 1). Therefore, we can ensure the repeatability or selection frequency of the final 16 features.

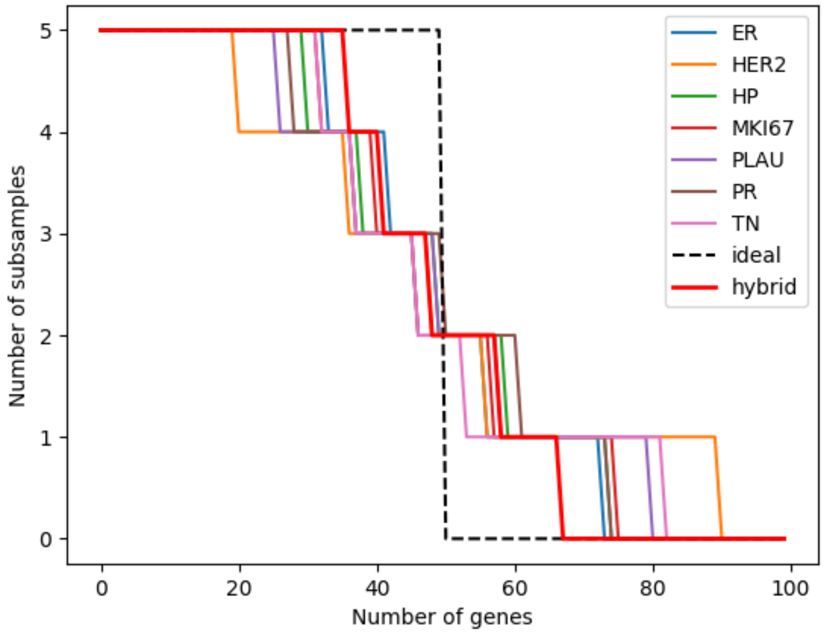

Fig. S7. Gene feature selection frequency plot

J. Independent validation test performance

From Table 1 in [2], we chose GSE21653 as the independent validation dataset, which records labels for disease-free survival (DFS). However, since certain clinical features are missing, predictions were only made with microarray expression profiles. We focus on the patients with DFS events and categorized those who survived less and more than 5 years (60 months) as poor and good prognosis class patients, respectively. Consequently, 14 patients belong to the poor prognosis class, and 69 patients are in the good prognosis class. We trained the support vector machine (SVM), random forest (RF), and microarray deep neural network (DNN) classifiers with the optimal hyper-parameters as detailed in Tables S4-6. These models were then tested on the independent validation dataset. The results were summarized in Table S8. We observe that the final 16 gene biomarkers are quite robust. Their prediction power is sufficient as all models still perform decently in stratifying breast cancer patients with DFS events in the independent validation dataset.

	GSE21653	
	ACC	AUC
SVM	0.6627	**0.7629**
RF	**0.8072**	0.7091
DNN	0.6747	0.7340

Table S8. Test performance of the independent validation GEO dataset

References

[1] Sehhati, M., Mehridehnavi, A., Rabbani, H., & Pourhossein, M. (2015). Stable gene signature selection for prediction of breast cancer recurrence using joint mutual information. IEEE/ACM transactions on computational biology and bioinformatics, 12(6), 1440-1448.

[2] Yan, Z., Wang, Q., Sun, X., Ban, B., Lu, Z., Dang, Y., ... & Guo, X. (2019). OSbrca: a web server for breast cancer prognostic biomarker investigation with massive data from tens of cohorts. Frontiers in oncology, 9, 1349.
